# Post-craniotomy coronal suture diastases in a transformed lower grade glioma

**DOI:** 10.1093/jscr/rjae004

**Published:** 2024-01-24

**Authors:** Teresa Morais Pinheiro, Mariam Awan, Oliver Wroe-Wright, Prajwal Ghimire, Francesco Marchi, Ali Elhag, José Pedro Lavrador

**Affiliations:** Departamento de Neurocirurgia, Hospital São José, Centro Hospitalar Lisboa Central, Lisboa 1150-199, Portugal; Neurosurgical Department, King’s College Hospital Foundation Trust, London SE5 9RS, UK; Neurosurgical Department, King’s College Hospital Foundation Trust, London SE5 9RS, UK; Neurosurgical Department, King’s College Hospital Foundation Trust, London SE5 9RS, UK; School of Biomedical Engineering and Imaging Sciences, King’s College London, London SE5 9RS, UK; Neurosurgical Department, King’s College Hospital Foundation Trust, London SE5 9RS, UK; Department of Neurosurgery, Neurocenter of Southern Switzerland, Ente Ospedaliero Cantonale, Lugano 6903, Switzerland; Neurosurgical Department, King’s College Hospital Foundation Trust, London SE5 9RS, UK; Neurosurgical Department, King’s College Hospital Foundation Trust, London SE5 9RS, UK

**Keywords:** craniotomy, coronal suture diastasis, transformed lower grade glioma

## Abstract

Cranial suture diastases are an uncommon clinical entity, with post craniotomy diastases being a previously undescribed finding in literature to our best knowledge. Herein, we report a case of a 28-year-old adult who underwent a second-stage low-grade glioma surgery 7 months after initial surgery. This study presents coronal suture diastases adjacent to the previously performed craniotomy. After literature and pathophysiology review, we found it to be unique and that the craniotomy can resemble the mechanical stress of trauma.

## Introduction

Cranial suture diastases are a rare entity across populations. Evidence in adult population is predominantly scarce. Scientific reports have described cases associated with trauma, namely epidural hematoma [1–3]. This entity is almost exclusively associated with the pediatric population due to a lack of fully ossified skull and more commonly attributed to trauma [2, 4] or increased intracranial pressure [5], such as in hydrocephalus or tumours. In this specific population, studies related with trauma have reported sutures diastasis associated high disruptive force injuries and more severe outcomes and complications [2]. In pediatric patients, traumatic suture diastasis is a common type of bone fracture due to the flexibility of the maturing bone. Depending on the age and force of the associated trauma, suture diastasis can be the only sign of the insult. The diastasis can occur when a linear fracture crosses the suture [4]. In the adult population, its frequency is even scarcer, with few evidence published.

The symptoms and presentation are related to the underlying cause, being most frequently headache, nausea and vomiting or decreased level of consciousness. In pediatric patients altered head conformation on physical examination is more likely to be noticeable than in adult population [5]. Treatment is directed to the underlying cause rather than the suture diastase itself, as in hematoma drainage, cerebrospinal fluid diversion procedures or tumour excision [1–6].

In this study, we present a case of post-craniotomy suture diastases in a 28-year-old adult.

## Case report

**Figure 1:**
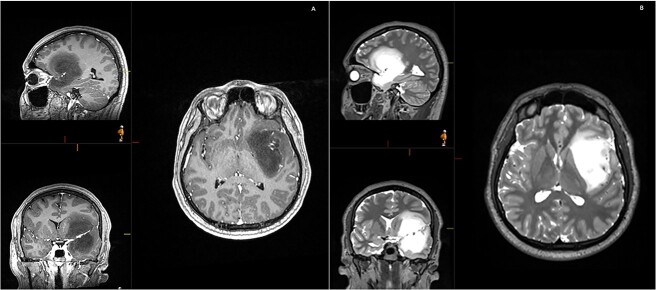
Initial MRI demonstrating a non-contrast enhancing left frontotemporal insular lesion. (A) T1-weighted post contrast; (B) T2-weighted.

A 28-year-old right-handed man, with unremarkable past medical history, was admitted at the Emergency Department with generalized tonic-clonic seizure with complete post-ictal recovery. The imaging revealed a left frontotemporal insular lesion without contrast enhancement (Fig. 1). A first stage asleep-awake-asleep craniotomy was performed with no complications. The patient remained clinically well throughout admission without neurological deficit. Histology revealed Isocitrate dehydrogenase-mutant grade 2 astrocytoma. Post-operative MRI confirmed residual tumour (approximately 30%), leading to planification of a second-stage surgery.

**Figure 2:**
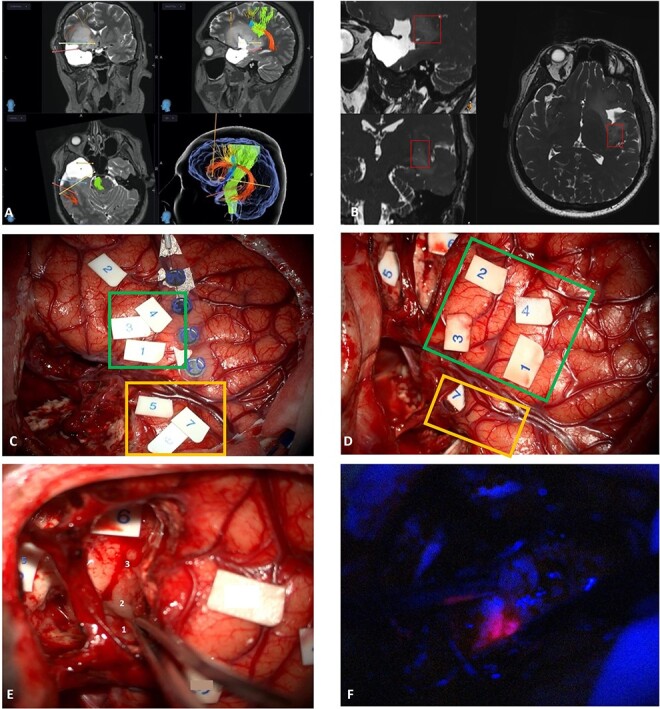
(A and B) Pre-operative planning. Tractography (A)—green: corticospinal tract; orange = Aacuate fasciculus; yellow = FST; blue = FAT) and visualization of perforator arteries and Virchow-Robin spaces (B). (C and D) Comparison between functional mapping between August 2022 (C) and March 2023 (D) (green box—stability of language eloquence in inferior frontal gyrus; yellow box—neuroplasticity at temporal level). (E and F) Surgical resection with origin of perforators from M1 [1], tumour surrounding perforators [2] and perforators [3]. Phonemic errors with high frequency stimulation [5], perforator’s territory [6] and 5-ALA enhancement (F).

Seven months later, the second-stage awake left frontotemporal craniotomy for resection of low-grade astrocytoma was performed to extend the resection towards the eloquent margins. Surgery was performed using 5-ALA, IONM and preoperative mapping (Fig. 2).

**Figure 3:**
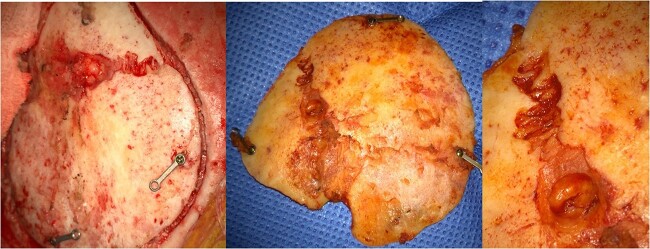
Bone flap with coronal suture diastases.

Intraoperatively, after incision with re-opening of previous reverse question mark incision at the left frontotemporal region and exposure of the previous craniotomy borders, a diastasis of the coronal suture in the bone flap was observed (Fig. 3).

Resection was performed through a transopercular approach, with frontal and temporal windows created. M1 and perforator arteries were identified with a cuff of tumour left at the origin of the perforators, limiting the resection medial and deeper to this point. Anterior to this point, resection was performed until the patient developed phonemic disturbances. Contrast enhancing tumour that was 5-ALA positive was resected and sent separately for analysis (Fig. 2). A comparison between the functional mappings between August 2022 and March 2023 was performed, revealing neuroplasticity at temporal level (Fig. 2).

**Figure 4:**
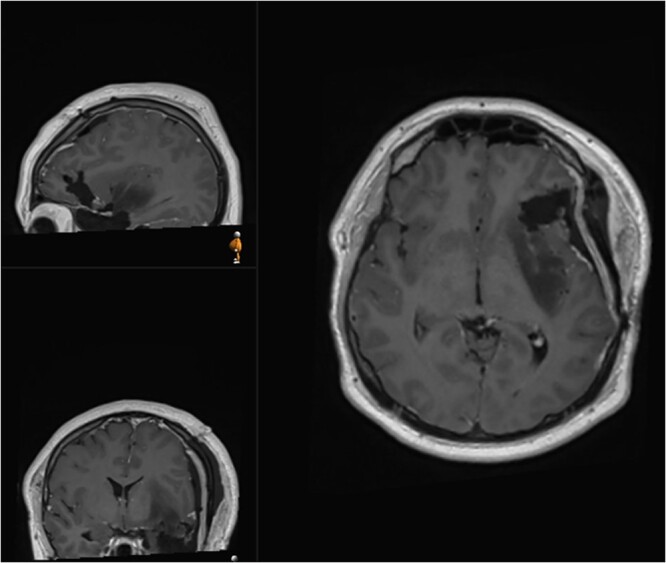
Postoperative (second surgery) MRI demonstrating residual tumour at the posterior margin of the insula.

The patient was discharged at day 3 after surgery with no neurological deficits. Post-operative MRI revealed removal of the contrast enhancing portion of the tumour and intentional residual tumour at the posterior margin of the insula (Fig. 4). Histology analysis revealed malignant transformation with IDH-mutant anaplastic astrocytoma, with malignant transformation (84% MGMT methylated, ATRX mutant, p53 overexpressed >90), WHO grade 3. The patient proceeded with oncological treatment in the form of chemotherapy and radiotherapy.

## Discussion

This case report illustrates a unique entity with no related published evidence. Our hypothesis is that the mechanical force associated with the trephination and fashioning of the craniotomy across the cranial sutures reproduced the effects of external trauma, as reported in the literature, creating a cranial suture diastasis.

Cranial suture closure is considered to be ‘normal’ at 2 months of age in the posterior fontanelle and at 5–24 months of age in the anterior fontanelle. Early closure of the anterior fontanelle can be due to craniosynostosis or microencephaly. Late closure can be associated with elevated intracranial pressure, malnutrition, hypothyroidism, Down Syndrome or achondroplasia [6]. In the light of craniosynostosis physiopathology, the early ossification of cranial suture, the standard of treatment is open or endoscopic-assisted suturectomy. We postulate that this principle might be related to the mechanism by which craniotomy-associated cranial suture diastasis is formed.

The lack of evidence and epidemiologic data about this clinical finding might be related to underreporting and previously scare numbers of reoperations in glioma surgeries.

Regarding recurrent glioblastomas, reoperation rates vary widely, based mostly on KPS at recurrence diagnosis. Yang *et al.* describe 13.9% reoperations [7], Goldman *et al.* report a reoperation rate of 54% [8] and Filippini *et al.* describes 25% of reoperations [9].

In low-grade glioma recurrence, reoperation rates are mostly related to extent of resection and residual tumour volume at time of first surgery [10], with Ribeiro *et al* reporting a 16.2% reoperation rate. As surgical techniques expand and adjuvant treatment options widen, survival outcomes improve [11], rendering reoperations more likely and, eventually, more frequent evidence of cranial suture diastasis.

It is described that cranial sutures’ ossification progresses with age [12]. This fact might justify that craniotomy-associated cranial suture diastasis can be more frequently encountered in a younger rather than older population due to progressively ossified cranial sutures.

## Conclusion

Our study is relevant in both broadening the understanding of cranial suture diastasis’ physiopathology and in raising further awareness in reoperation cases to adjust patient counselling (and eventually avoid cranial suture crossing at first-time surgery whenever possible).
